# KIF20A activated by transcription factor GATA2 promotes cell growth in hepatitis B virus-related hepatocellular carcinoma

**DOI:** 10.3389/fcimb.2024.1497427

**Published:** 2024-11-18

**Authors:** Juan Xu, Wenhua Cheng, Yi Wang, Yunpeng Zhou, Zhiming Wang, Yunyan Dai, Yaoxuan Li, Pinggui Chen, Ting Liu, Yifan Li, Gaopeng Li, Wenqing Qu, Jing Chen

**Affiliations:** ^1^ General Surgery Department, Shanxi Bethune Hospital, Taiyuan, Shanxi, China; ^2^ Department of Gastroenterology, Shanxi Cancer Hospital, Taiyuan, Shanxi, China; ^3^ General Surgery Department, Third Hospital of Shanxi Medical University, Taiyuan, Shanxi, China; ^4^ School of Public Health, Shanxi Medical University, Taiyuan, Shanxi, China; ^5^ Department of Hepatobiliary, Pancreatic and Gastrointestinal Surgery, Shanxi Cancer Hospital, Taiyuan, Shanxi, China; ^6^ Department of breast surgery, Shanxi Cancer Hospital, Taiyuan, Shanxi, China

**Keywords:** hepatocellular carcinoma, hepatitis B virus, apoptosis, prognosis, HBx protein

## Abstract

**Background:**

Elevated evidence suggests that KIF20A plays an important role in hepatocellular carcinoma (HCC) progression. Nevertheless, the underlying mechanism by which KIF20A promotes HCC cell growth are not well understood.

**Methods:**

Using TCGA-LIHC RNAseq and GEO datasets, we assessed the KIF20A expression and patient survival in HCC and hepatitis B virus (HBV)-related HCC. Mutant and CNV analysis were performed to evaluate the genetic alteration of KIF20A in HCC. PPI network and GSEA enrichment was utilized for analyzing the KIF20A-related genes and involved pathways in HCC. To further explore regulatory mechanism in HBV-related HCC, PROMO prediction and luciferase reporter system was utilized for verifying HBx/GATA2/KIF20A binding sites. CCK-8 and flow cytometry were carried out to determine the regulation of GATA2-KIF20A on HBV-related HCC cell proliferation and apoptosis.

**Results:**

KIF20A was significantly upregulated in pan-cancer (including HCC). KIF20A mRNA level was a significant independent predictor of overall survival in HBV-related HCC patients. Genetic alterations analysis revealed the copy number gain and amplification triggered KIF20A upregulation in HCC. In addition, the genes associated with KIF20A expression in HCC was enriched in PLK1 pathway and cell cycle in HCC. HBx might indirectly binds to KIF20A promoter via regulating GATA2. Additionally, transcription factor GATA2 directly binds to the promoter region of KIF20A. Overexpression of GATA2 promotes HepG2.2.15 cell growth and inhibits cell apoptosis via modulating KIF20A.

**Conclusions:**

Our findings demonstrated that HBx contributed to cell proliferation by interacting with GATA2 and KIF20A in HBV-related HCC.

## Introduction

Hepatocellular carcinoma (HCC) is the most common type of primary liver cancer, accounting for the majority of liver cancer cases worldwide (Forner et al., 2018). HCC is a highly aggressive and malignant tumor that arises from hepatocytes (Forner et al., 2018). Due to high incidence and mortality rates, it makes HCC as a pressing public health issue, with approximately 905,700 new cases and 830,200 deaths worldwide in 2020 (Sung et al., 2021). Chronic infection with hepatitis B virus (HBV) is the leading risk factor for HCC, and it is estimated that up to 80% of HCC cases are attributable to viral infections (Okuda, 2000; Wang et al., 2017). The prognosis for HBV-related HCC patients remains unfavorable, with a 5-year survival rate of less than 20%, despite advances in diagnostic and therapeutic strategies (Befeler and Di Bisceglie, 2002; Levrero and Zucman-Rossi, 2016). Therefore, there is a critical need for understanding the regulatory mechanism by which modulates the development and progression of HBV-related HCC, and to identify novel therapeutic targets for HCC treatment.

Kinesin family member 20A (KIF20A) is a member of the kinesin superfamily, a group of molecular motors which are vital for numerous cellular functions, such as cell proliferation, vesicular transport, and cancer progression (Mandal et al., 2019; Jin et al., 2023). KIF20A is emerging as an oncogene, being widely expressed in tumor tissues and potentially contributing to cell growth and tumor metastasis by interacting with JAK-STAT3 pathway in colorectal cancer and glioma (Johnson et al., 2018; Xiong et al., 2019). In HCC, accumulation of KIF20A promotes the pathologic hepatocyte proliferating and tumorigenic potential (Gasnereau et al., 2012). Furthermore, KIF20A contributes to tumor resistance to chemotherapy in HCC, highlighting its potential as a therapeutic target for HCC treatment (Wu et al., 2021). Considering the distinct molecular characteristics between HCC and HBV-related HCC, as the integration of HBV DNA into the host genome and activation of oncogenic pathways, this research focused on the molecular mechanism regulating KIF20A in HBV-related HCC.

To elucidate the role of KIF20A in HBV-related HCC, we performed a comprehensive analysis of its expression and genetic alterations using TCGA-LIHC and GEO datasets. Furthermore, we explored the cellular effects of KIF20A and its upstream transcription factor GATA2 on cell growth and apoptosis in an HBV-infected HCC cell line, aiming to elucidate the intricate relationship between KIF20A and HBV infection in HCC tumor progression.

## Materials and methods 

### TCGA-LIHC and GEO data preprocessing

RNA sequencing data and clinical features of hepatocellular carcinoma (HCC) patients were obtained from TCGA database. The R software was utilized to translate the RNA matrix data. A search was conducted on the GEO database utilizing the keywords “hepatocellular carcinoma” and “hepatitis B virus” to identify relevant datasets. The search results were filtered based on the following criteria: (1) human HCC tissues and non-tumor tissues, (2) transcription expression data, and (3) availability of patients’ survival data. The GSE19665, GSE135631, and GSE14520 datasets were selected for further analysis using R packages.

### Expression patterns and survival value of KIF20A in HCC

The differential expression of KIF20A at the mRNA level between tumor tissues (including pan-cancer and HCC) and corresponding non-tumor tissues was assessed using data from TCGA and GEO datasets. Additionally, KIF20A protein expression in HCC tissues was examined via immunohistochemistry (IHC) in the Human Protein Atlas (HPA, https://www.proteinatlas.org/). Furthermore, the association between KIF20A mRNA levels and clinical characteristics was explored through analyzing the TCGA-LIHC data. The correlation between KIF20A mRNA expression and survival outcomes in HCC, including OS, DS, and PFI, was evaluated using Cox regression survival analysis with the R package “survival”.

### PPI network construction and GSEA enrichment analysis

KIF20A-related genes were identified using the LinkedOmics database by applying a filter of |Pearson’s correlation| >= 0.5 and p < 0.05. Similarly, the cBioPortal database was used to screen for KIF20A-associated genes with |Spearman’s correlation| >= 0.5 and p < 0.05. The common KIF20A-related genes identified by LinkedOmics and cBioPortal databases were then subjected to construct a PPI network via STRING. Gene Ontology (GO) and KEGG pathway enrichment analyses were conducted using the DAVID database. To explore the potential biological roles and key functions of signature genes in HCC, GSEA was carried out with a threshold of |NES|> 1, q < 0.25, and p < 0.05.

### Cancer cell lines and cell culture

HepG2 cells were obtained from the American Type Culture Collection (ATCC, USA). Huh-2 and HepG2.2.15 cells were sourced from the National Collection of Authenticated Cell Cultures (China). All cell lines were maintained in base medium supplemented with 10% fetal bovine serum (FBS, Catalog Number: A5670701, Gibco, USA) and cultured in a humidified atmosphere at 37 °C and 5% CO_2_. To ensure the absence of mycoplasma contamination, cells were tested every 3 months using the Mycoplasma PCR Detection Kit (Catalog Number: ab289834, Abcam, UK).

### Plasmid construction and cell transfection

The plasmids encoding HBx or GATA2 were constructed by integrating the synthetically produced cDNA into the pcDNA3.1 vectors (Catalog Number: V79020, Invitrogen, USA) via the EcoRI/XbaI MCS. To generate silencing RNAs to knockdown GATA2 or KIF20A, the following sequences are synthesized: si-GATA2 (forward 5’-AGA AAA AGG AUG UAU UUA CAG-3’, reverse 5’-GUA AAU ACA UCC UUU UUC UGC-3’), si-KIF20A (forward 5’-UUC UAA UAG GUC AUA AAG CAG-3’, reverse 5’-GCU UUA UGA CCU AUU AGA ACC-3’). siRNAs or vectors were transfected into HepG2.2.15 cells using Lipofectamine 3000 Reagent (Catalog Number: L3000150, Invitrogen, USA).

### Quantitative real-time PCR

Total RNA was isolated from HCC cells utilizing TRIzol reagent (Catalog Number: 15596018CN, Invitrogen, USA) and then transcript into cDNA with the Reverse Transcription Kit (Catalog Number:4368813, Applied Biosystems, USA). cDNA was subsequently used for quantitative RT-PCR analysis with gene-specific primers and SYBR Green Mix (Catalog Number: 4309155, Applied Biosystems, USA). The primers for qPCR: human GATA2 (forward 5’-CCG CCA CAT CCA TCC TAG C-3’, reverse 5’-CTG CGA GTC GAG GTG ATT GA-3’), human KIF20A (forward 5’-CAA GGG ATC CTT TCT CCG CC-3’, reverse 5’-GAA CCT GCT GCT TGT CCT CT-3’), GAPDH (forward 5’-TTT TGC GTC GCC AGC C-3’, reverse 5’-ATG GAA TTT GCC ATG GGT GGA-3’). Relative mRNA expression levels were determined by normalizing the Ct-values of the target genes to GAPDH and applying the ΔΔCt method.

### Dual luciferase reporter gene assay

24-well plates were plated with HEK-293T cells. Until the cells grow reaching 70% confluence, cells were transfected with 0.75 μg of pGL3-basic/pGL3-basic-KIF20A luciferase reporter plasmid, along with 0.25 μg of overexpression vectors. After 48 hours post-transfection, luciferase activity was measured using Pierce Firefly Luciferase Glow Assay Kit (Catalog Number: 16176, Thermo Fisher, USA). More precisely, the cells were rinsed once with PBS buffer and resuspended in cell lysis buffer. Then the cell plate was shaken on a shaker platform at moderate speed for 15 minutes. Each well was added with 30 µl of cell lysate and 50 µl of working solution. After 10 minutes, light output was detected using luminometer. Firefly luciferase activity was normalized to the activity of the pRL-TK reporter gene.

### Cell Counting Kit (CCK-8) assay

CCK-8 assay was performed to assessing cell proliferation after 48 hours of transfection. HepG2.2.15 cells were harvested, resuspended with PBS buffer, and plated in a 96-well plate at a density of 5 × 10^^3^ cells per well. The cells were incubated for 24, 48, 72, and 96 hours, followed by adding 10 μL of CCK-8 reagent (Catalog Number: CA1210, Solarbio, China) to each well. After a 2-hour incubation, the absorbance of 450 nm was measured.

### Flow cytometry

Quantification of apoptotic percentage was performed by flow cytometry utilizing an Annexin V-APC/PI staining kit (Catalog Number: V13242, Invitrogen, USA). After transfection, HCC cells were trypsinized and washed with cold PBS. The resuspend cells in 100 μL of Annexin V binding buffer was stained with Annexin V-APC (concentration: 1 μg/mL), and then incubated for 15 minutes at room temperature in the dark. After adding 5 μL of propidium iodide (PI) (concentration: 50 μg/mL), the cell suspension was incubated for an additional 5 minutes. BD FACSCalibur was utilized to perform flow cytometry analysis. FlowJo software (version 10) was employed to analyze apoptosis data.

### Statistical analysis

All experiments were carried out in a minimum of three biological replicates. Statistical analysis was performed using the two-tailed Student’s t-test in GraphPad Prism 8 (San Diego, CA), with a p-value of less than 0.05 considered statistically significant.

## Results

### KIF20A expression and clinical outcome in HCC tissues via analyzing TCGA cohorts

**Figure 1:**
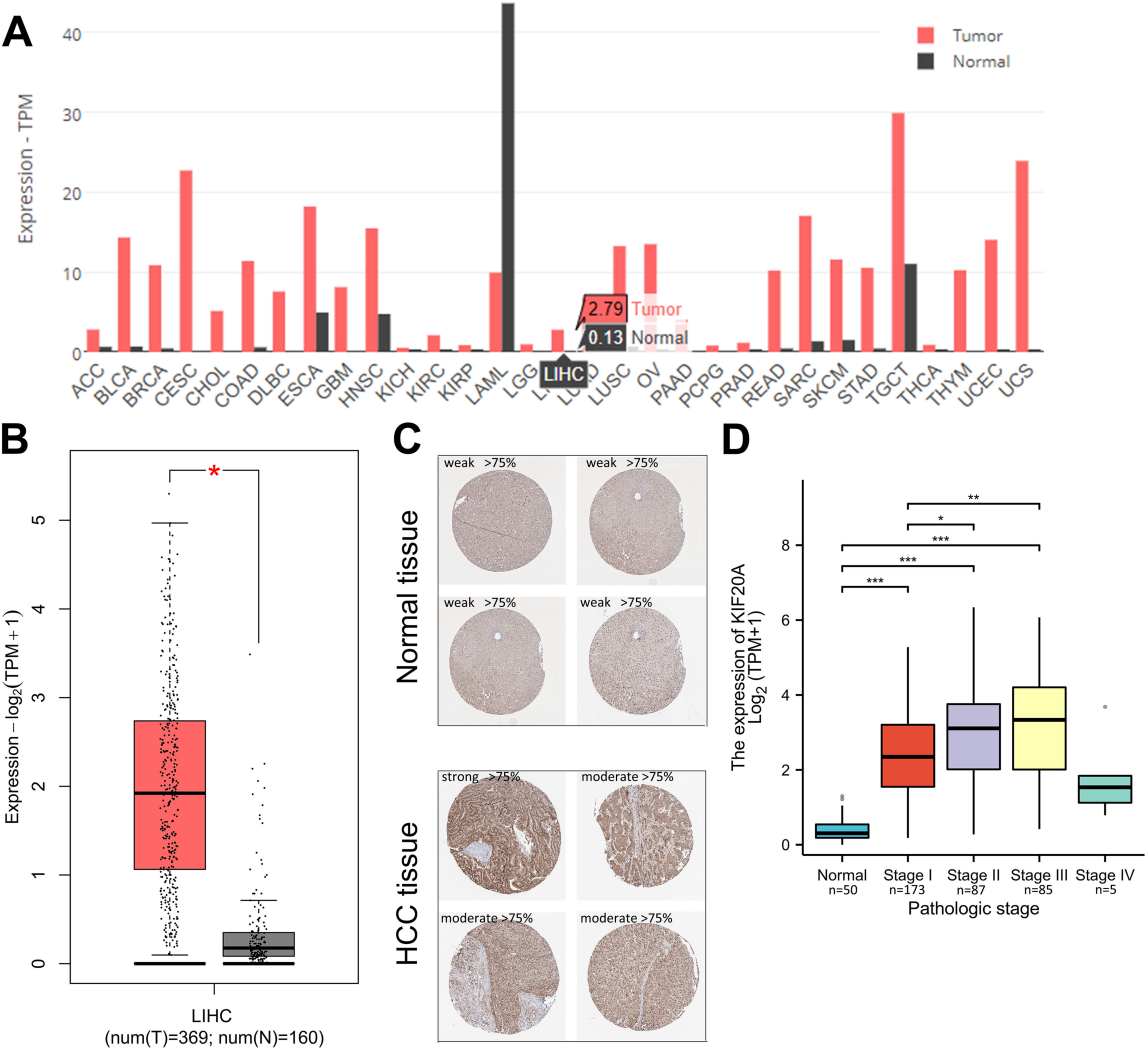
TCGA analysis of KIF20A expression in HCC tissues **(A)** KIF20A expression levels in pan-cancer. **(B)** Box plots showing the mRNA KIF20A expression levels in HCC tissues and normal samples. **(C)** Representative immunohistochemical images displaying KIF20A staining in HCC tumors and normal liver tissues. **(D)** mRNA expression levels of KIF20A in different HCC pathologic stages. * p < 0.05, ** p < 0.01, *** p < 0.001.

To elucidate the involvement of KIF20A in the development of human cancers, we performed a pan-cancer analysis of KIF20A expression using the TCGA dataset, which provides a potential resource for exploring the molecular characteristics of diverse cancer types. In the TCGA cohorts, we noticed an extensive enrichment of KIF20A in pan-cancer (**Figure 1A**). As depicted in **Figures 1B, C**, KIF20A expression was low in normal samples, but KIF20A mRNA and protein was significantly up-regulated in LIHC tissues. In addition, HCC patients with high histologic grade (p < 0.001) or advanced pathologic T stage (p = 0.002) had further upregulated KIF20A expression levels (**Table 1**). RNA-sequencing analysis in human HCC tissues showed that while KIF20A mRNA is barely expressed in normal liver samples, HCC patients with stage I/II/III have ∼6.08-fold, ∼7.50-fold and ∼8.02-fold higher expression levels of KIF20A (p < 0.0001) (**Figure 1D**).

**Table 1:** The baseline data of clinical HCC patients in TCGA.

Characteristics	Low expression of KIF20A	High expression of KIF20A	P value
n	187	187	
Gender, n (%)			0.097
Female	53 (14.2%)	68 (18.2%)	
Male	134 (35.8%)	119 (31.8%)	
Age, n (%)			0.011
<= 60	76 (20.4%)	101 (27.1%)	
> 60	110 (29.5%)	86 (23.1%)	
Histologic grade, n (%)			**< 0.001**
G1	38 (10.3%)	17 (4.6%)	
G2	100 (27.1%)	78 (21.1%)	
G3	44 (11.9%)	80 (21.7%)	
G4	3 (0.8%)	9 (2.4%)	
Pathologic T stage, n (%)			**0.002**
T1	109 (29.4%)	74 (19.9%)	
T2	39 (10.5%)	56 (15.1%)	
T3	32 (8.6%)	48 (12.9%)	
T4	4 (1.1%)	9 (2.4%)	
Pathologic N stage, n (%)			1.000
N0	121 (46.9%)	133 (51.6%)	
N1	2 (0.8%)	2 (0.8%)	
Pathologic M stage, n (%)			0.563
M0	128 (47.1%)	140 (51.5%)	
M1	3 (1.1%)	1 (0.4%)	

p < 0.05 (bold values) is considered as statistically significance.

**Figure 2:**
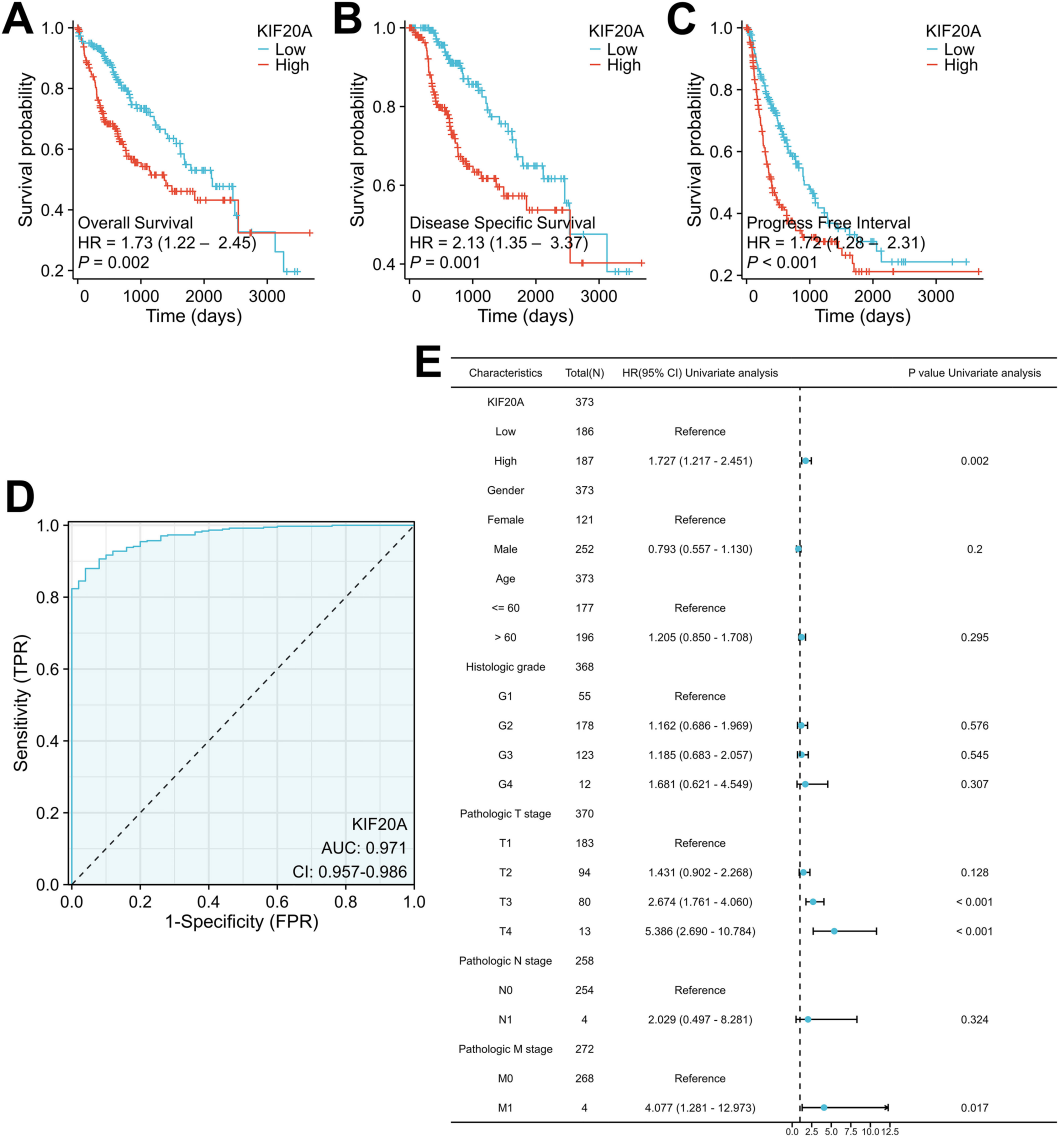
High KIF20A expression occurs in HCC tissues in association with worse clinical outcome **(A-C)** Kaplan–Meier analysis of OS in the TCGA-LIHC cohort with respect to KIF20A expression levels. **(D)** ROC curve analysis to assess the performance of biomarker KIF20A expression panel in a TCGA-LIHC cohort. **(E)** Forest plot of the multivariable Cox regression analysis for clinical outcome in the TCGA-LIHC cohort studied for KIF20A expression level and other prognostic factors.

Next, we investigated the relationship between KIF20A levels and survival in HCC patients. Our results indicated that high KIF20A expression was obviously associated with worse OS (log-rank p = 0.002; HR = 1.73, 95% CI = 1.22-2.45), DSS (log-rank p = 0.001; HR = 2.13, 95% CI = 1.35-3.37), and PFI (log-rank p < 0.001; HR = 1.72, 95% CI = 1.28-2.31) in the TCGA-LIHC cohort (**Figures 2A–C**). We also evaluated the predictive value of KIF20A expression utilizing ROC curve analysis, which demonstrated an AUC of 0.971 (95% CI: 0.957-0.986) (**Figure 2D**). To evaluate the potential of KIF20A expression as an independent biomarker, we conducted a multivariate analysis of KIF20A levels and clinical characteristics in the TCGA-LIHC cohort. Notably, Cox regression analysis revealed that KIF20A level was a significant independent predictor of OS (HR = 1.727, 95% CI = 1.217-2.451, p = 0.002) (**Figure 2E**), DSS (HR = 2.135, 95% CI = 1.352-3.371, p = 0.001), and PFI (HR = 1.720, 95% CI = 1.282-2.30, p < 0.001) (**Supplementary Figure S1**).

### KIF20A expression and clinical outcome in HBV-related HCC tissues via analyzing GEO datasets

**Figure 3:**
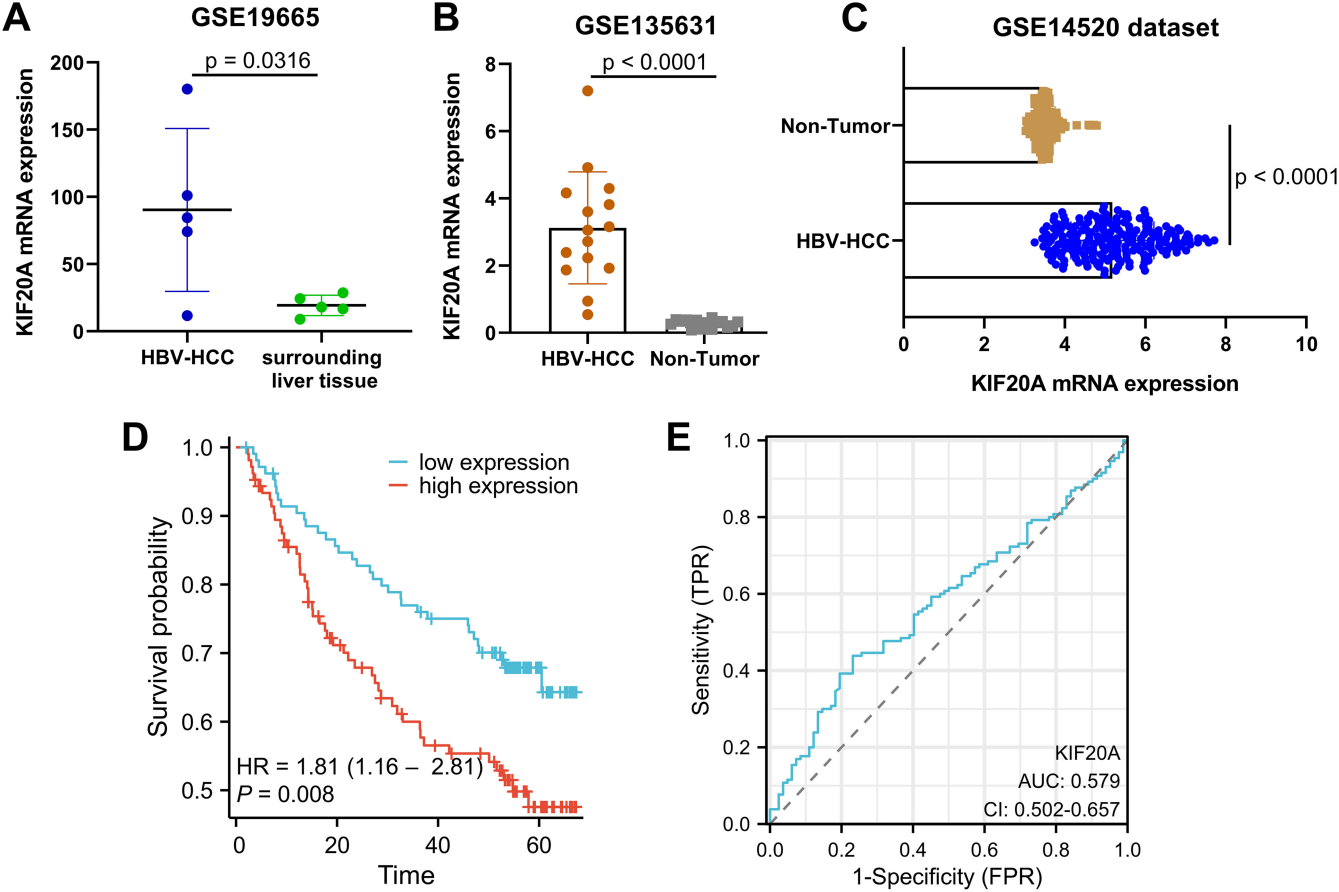
KIF20A overexpression drives poor prognosis of patients with HBV-related HCC **(A-C)** KIF20A mRNA expression levels in GSE19665, GSE135631 and GSE14520 datasets containing HBV-related HCC and normal liver samples. **(D)** Kaplan-Meier survival curve representing OS of patient with HBV-related HCC based on circulating KIF20A levels. **(E)** A ROC curve analysis to compare the performance of KIF20A model in GSE14520 dataset.

Given the results described above, we sought then to elucidate the involvement of KIF20A in HBV-related HCC tissues. To achieve it, we first screened out GSE dataset that contains HBV-related HCC samples and patients’ clinical information. In GSE19665 dataset, KIF20A mRNA expression was obviously higher in HBV-infected HCC cases (n = 5) than in surrounding liver tissues (n = 5) (p = 0.0316, **Figure 3A**). Additionally, KIF20A levels were upregulated ∼8.3-fold in HBV-related HCC tissues (n = 15) in comparation with non-tumor tissues (n = 15) in GSE135631 dataset (p < 0.0001, **Figure 3B**). To examine how transcription of KIF20A oncogene behaved under HBV infection, we acquired the GSE14520 dataset to analyze more HBV-related HCC samples. As depicted in **Figure 3C**, the HBV-HCC tissues (n = 212) showed a higher KIF20A mRNA level than non-tumor cases (n = 221) in GSE14520 dataset (p < 0.0001). GSE14520 dataset revealed that a higher KIF20A expression level associates to worse OS in HBV-related HCC patients (p = 0.008, **Figure 3D**). ROC curve was generated to determine the diagnostic ability of KIF20A. An AUC of 0.579 (CI = 0.502-0.657) was calculated, indicating the medium specific ability of KIF20A expression to predict the patients’ prognosis in 212 HBV-related HCC tissues (**Figure 3E**).

### KIF20A is highly amplified in HCC

**Figure 4:**
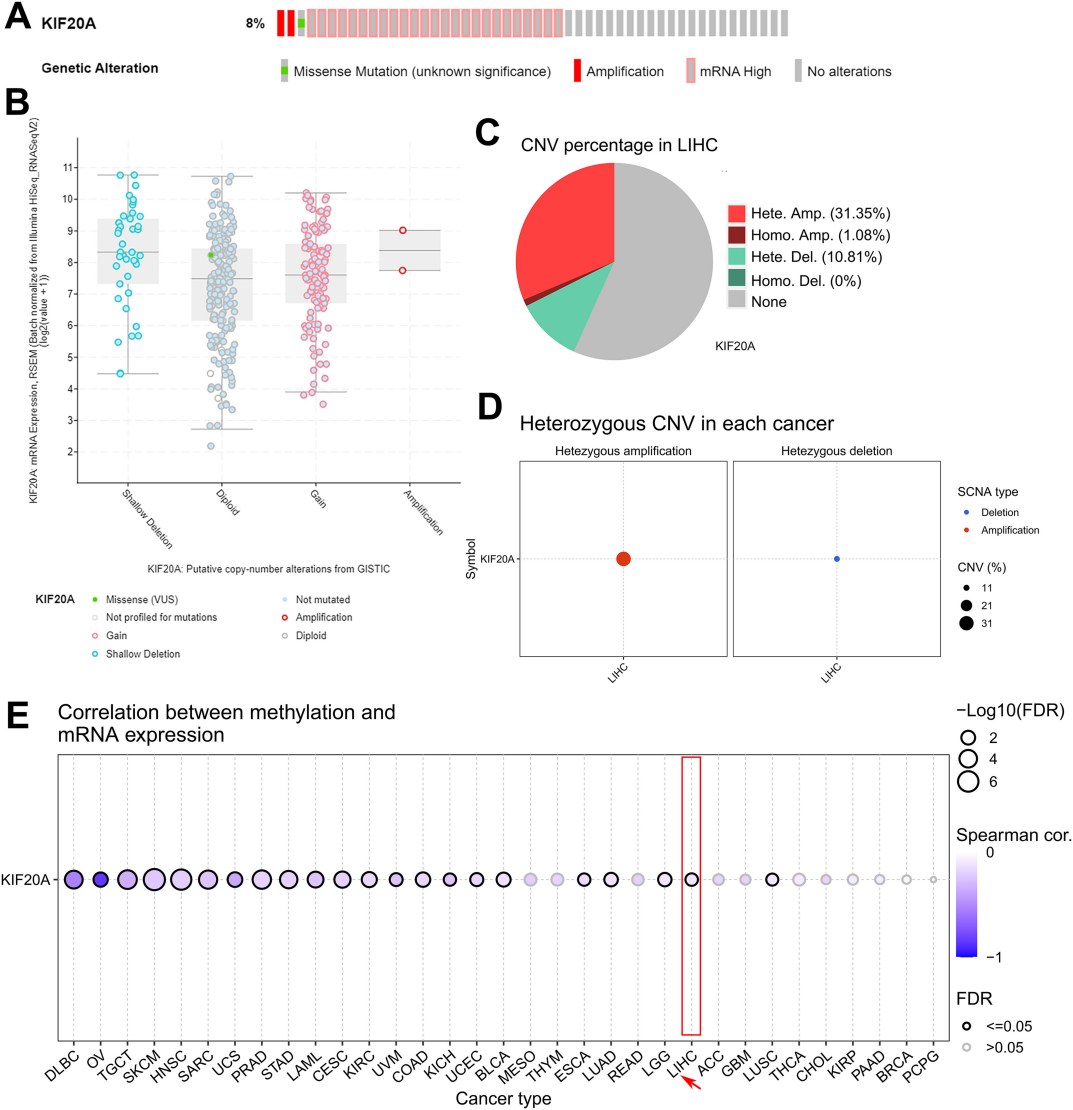
KIF20A is highly amplified in HCC **(A)** cBioportal mutation profile of KIF20A in HCC. **(B)** Box plot showing the correlation between KIF20A mRNA expression and CNV types in HCC. **(C, D)** A global profile and the constitute of the heterozygous/homozygous CNV of KIF20A gene in HCC. **(E)** Correlation between methylation and KIF20A expression in pan-cancer.

To characterize possible genetic or epigenetic alterations of KIF20A in tumors, we first studied in silico a set of pan-cancer via cBioportal database. The cBioportal genomics analysis revealed KIF20A amplification or high mRNA in 8% (348 cases) of HCC (**Figure 4A**). Up to a third of the KIF20A copy number gain (111 cases, 31.9%) or amplification (2 cases, 0.5%) occurred with gene alteration in HCC (**Figure 4B**). To validate structural variation in HCC, samples from patient with HCC was subjected to GSCA copy number variation (CNV) analysis. The pie plot exhibits us a global profile and the constitute of the heterozygous/homozygous CNV of KIF20A gene in HCC. As shown in **Figures 4C, D**, HCC tumor has 31.35% heterozygous amplification, 10.81% heterozygous deletion and 1.08% homozygous amplification in KIF20A gene. However, data-mining of the available profile of HCC geneset showed that KIF20A methylation was not associated with KIF20A mRNA upregulation (Spearman correlation = -0.11) (**Figure 4E**).

### Correlation analysis of KIF20A-related genes in HCC

**Figure 5:**
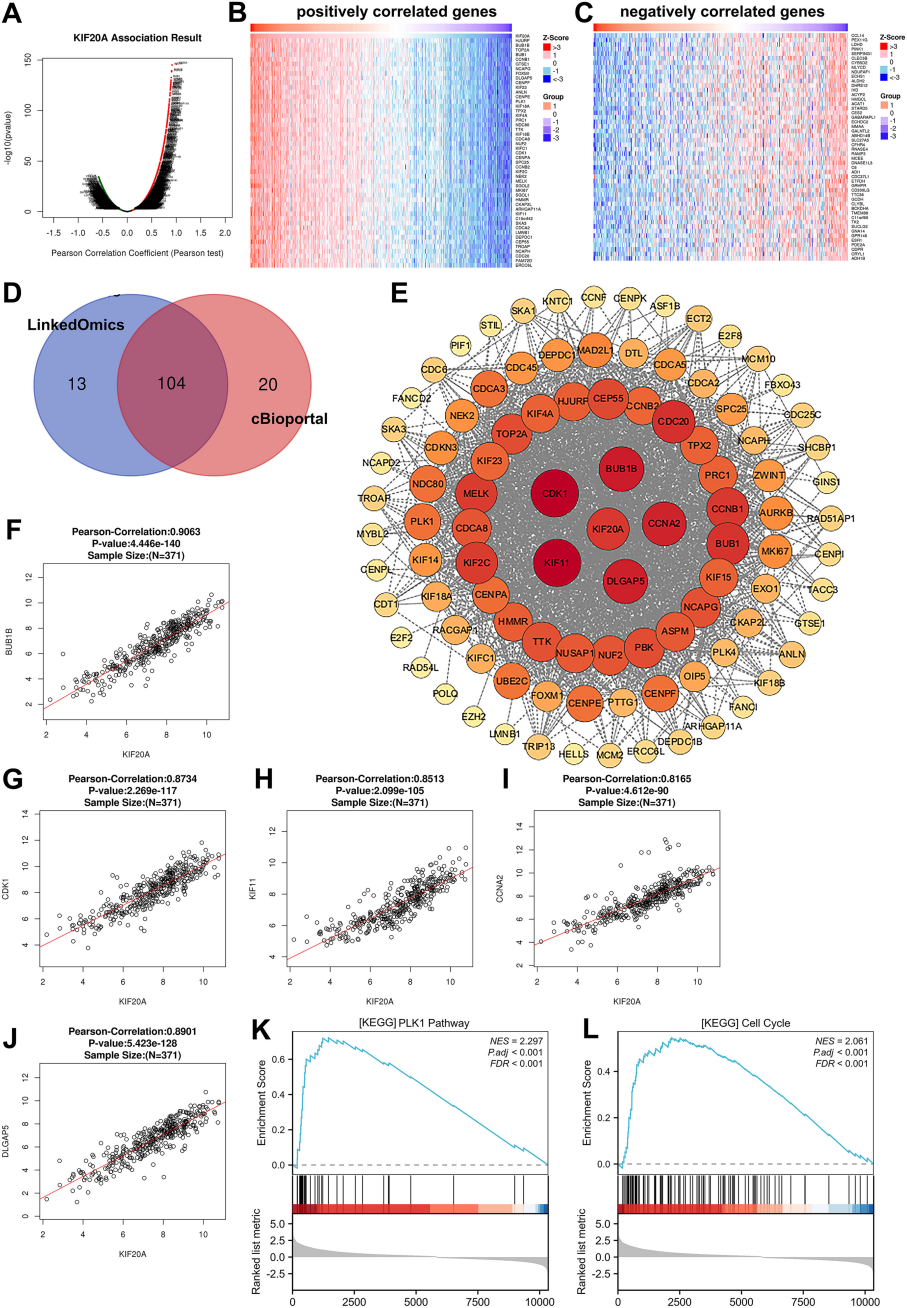
Correlation analysis of KIF20A-related genes in HCC **(A)** Volcano plot of all differentially expressed genes that related to KIF20A expression in HCC. **(B, C)** The top 50 positively correlated and negatively correlated gene. **(D)** Veen diagram showing the overlap of KIF20A-co-expressed genes in LinkedOmics and cBioportal. **(E)** The interaction between 104 KIF20A-co-expressed proteins was predicted by using STRING database and visualized by Cytoscope. **(F-J)** Correlation analysis between KIF20A expression and five KIF20A-co-expressed genes (BUB1B, CDK1 KIF11, CCNA2, DLGAP5). **(K, L)** Heatmap of enriched genes in PLK1 pathway and cell cycle via GSEA analysis.

To understand how KIF20A impacts essential gene expression to contribute to HCC tumor progression and clinical prognosis, we characterized changes to downstream mRNA levels in HCC. We performed the LinkedOmics analysis and showed that a total of 417 genes were correlated with KIF20A expression levels in HCC tissues, including 388 positively related genes and 29 negative related genes (|Pearson’s correlation|  >= 0.5, p  <  0.05) (**Figure 5A**). Heat map in **Figures 5B, C** shows the top 50 positively and negatively correlated genes. In addition, Spearman correlation analysis via cBioportal is also indicative of 463 genes that were related to KIF20A levels (|Spearman’s correlation|  >= 0.5, p  <  0.05). To screen out the more relevant genes in HCC samples, we applied a condition of higher correlation (>= 0.75) and overlapped common genes between cBioPortal and LinkedOmics (**Figure 5D**). We excavated the STRING database and conducted PPI analysis using the overlapped 104 KIF20A-coexpressed genes. Interestingly, we observed potential interactions between KIF20A and co-expressed genes. The minimum required interaction score in the PPI network is highest confidence (0.9). The PPI network shows the potential co-expressed proteins with KIF20A (p-value < 1.0e-16, **Figure 5E**). Moreover, GO and KEGG enrichment analysis indicated that KIF20A affected several biological process and pathways related to cell response, such as cell division and cell cycle (**Table 2**). As shown in **Figures 5F–J**, the mRNA levels of the top five co-expressed genes exhibited a significant positive correlation with KIF20A expression, suggesting a potential functional relationship between these genes in HCC (Pearson correlation >= 0.8, p < 0.001). Importantly, single gene expression and GSEA analysis provided evidence that KIF20A may be implicated in the PLK1 pathway and cell cycle in HCC, implying a potential role for KIF20A in the regulation of cellular processes and its possible involvement in the pathogenesis of HCC (**Figures 5K, L**).

**Table 2:** GO and KEGG pathway enrichment analysis for 104 KIF20A co-expressed genes.

Category	Term	Count	%	PValue	Fold Enrichment	FDR
BP_DIRECT	cell division	46	44.23	1.10E-50	23.57829	5.27E-48
BP_DIRECT	chromosome segregation	19	18.27	3.67E-23	36.0525	8.80E-21
BP_DIRECT	phosphorylation	11	10.58	0.001288	3.429289	0.015205
BP_DIRECT	microtubule-based movement	10	9.62	2.40E-10	24.6675	1.15E-08
BP_DIRECT	DNA repair	9	8.65	5.98E-05	6.65191	0.001149
BP_DIRECT	mitotic metaphase chromosome alignment	8	7.69	2.12E-09	35.88	8.49E-08
BP_DIRECT	cell cycle	8	7.69	0.001235	4.872593	0.0152
BP_DIRECT	attachment of mitotic spindle microtubules to kinetochore	7	6.73	6.60E-11	92.092	3.52E-09
BP_DIRECT	regulation of cyclin-dependent protein serine/threonine kinase activity	7	6.73	1.24E-07	29.39106	4.26E-06
BP_DIRECT	G2/M transition of mitotic cell cycle	7	6.73	2.05E-07	27.08588	6.16E-06
BP_DIRECT	DNA damage response	7	6.73	0.004514	4.499609	0.045138
KEGG_PATHWAY	Cell cycle	21	20.19	6.88E-24	25.5421	3.10E-22
KEGG_PATHWAY	Oocyte meiosis	10	9.62	2.48E-08	13.82546	5.59E-07
KEGG_PATHWAY	Motor proteins	10	9.62	5.00E-07	9.755021	7.50E-06
KEGG_PATHWAY	Progesterone-mediated oocyte maturation	8	7.69	1.25E-06	13.85037	1.40E-05
KEGG_PATHWAY	Cellular senescence	7	6.73	1.31E-04	8.568264	0.001179
KEGG_PATHWAY	Human T-cell leukemia virus 1 infection	7	6.73	8.62E-04	6.032365	0.006464

### HBx activates the KIF20A promoter by interacting with transcription factor GATA2 in HepG2.2.15 cells

**Figure 6:**
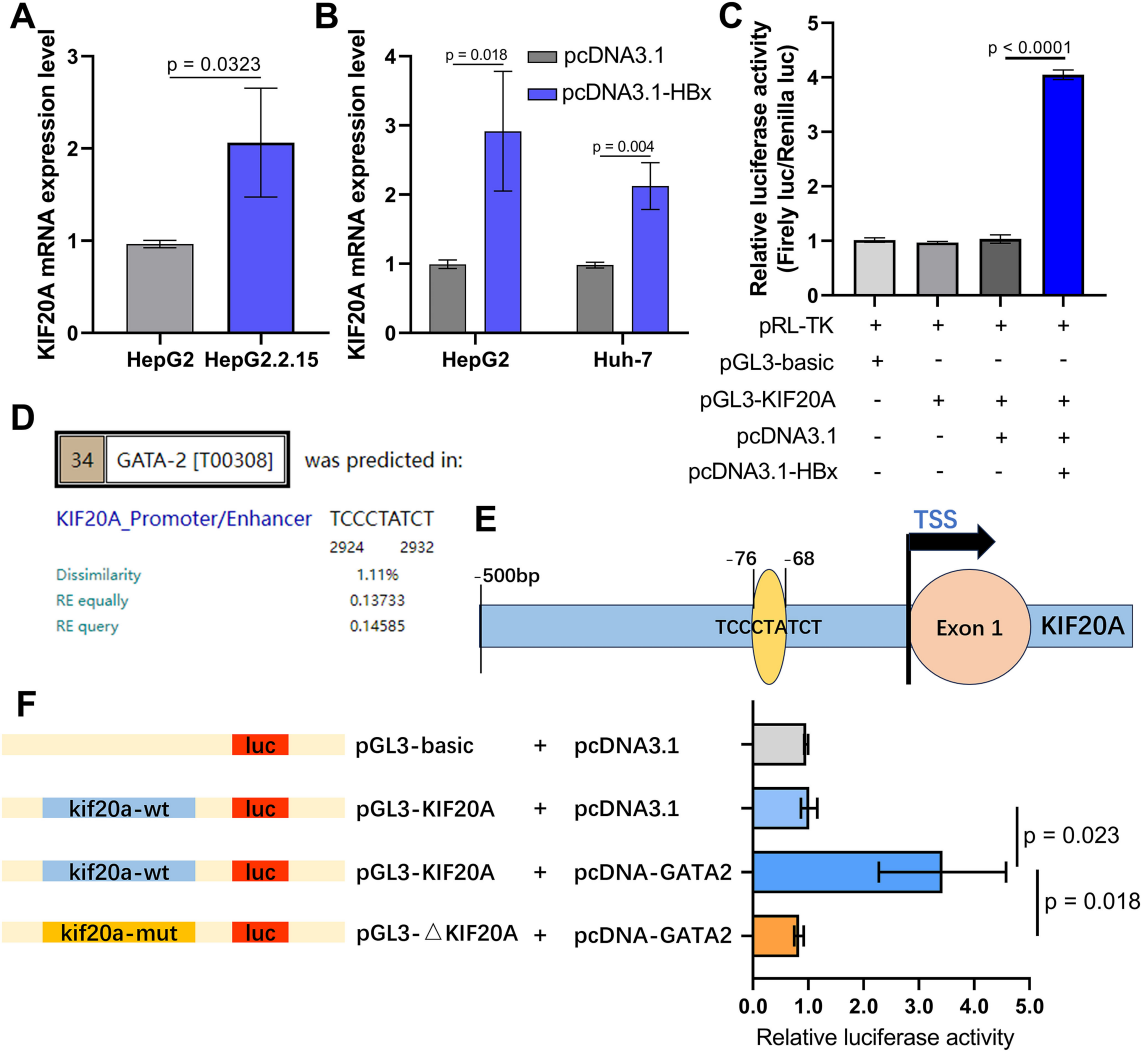
HBx protein enhances KIF20A promoter activity by forming a complex with the transcription factor GATA2 in HCC cells **(A)** qPCR detecting the KIF20A mRNA expression levels in HepG2 and HepG2.2.15 cell lines. **(B)** pcDNA3.1/pcDNA3.1-HBx was transfected into HepG2 and Huh-7 cells. The expression levels of KIF20A were assessed utilizing qPCR. **(C)** Luciferase activity of 293T cells transfected with a pGL3-basic-luc reporter or pGL3-KIF20A-luc reporter together with a plasmid expressing HBx. **(D)** PROMO prediction showing the binding ability between GATA2 and KIF20A promoter. **(E)** Schematic diagram of the KIF20A promoter region containing binding sites with GATA2. **(F)** Dual luciferase reporter assay was carried out to verify the binding of GATA2 and KIF20A promoter.

To verify the role of KIF20A in HCC cells, we first tested the KIF20A mRNA expression in HepG2 cell line (HBV negative) and HepG2.2.15 cell line (HBV positive). HBV infection in HepG2.2.15 cells significantly increased KIF20A expression at the mRNA level, indicating translational regulation of KIF20A by HBV (p = 0.0323, **Figure 6A**). Next, we transfected plasmids into HepG2 and Huh-7 cells to upregulate KIF20A expression levels (p = 0.018, **Figure 6B**) and detected the HBX-KIF20A binding on the KIF20A promoter. As shown in **Figure 6C**, luciferase reporter gene analysis suggested that HBx binds with the promoter site of KIF20A (p < 0.0001).

Established study reported that HBx is as a non-specific regulatory factor via interacting with other transcriptional factors, instead of directly binding to DNA in HCC (Andrisani and Barnabas, 1999). We focused our investigation to HBx-interacting transcription factors essential for HCC cells. For HCC cells, ChIP-chip assay has identified that HBx was enriched on the promoter of 144 transcript factors, including GATA2 (Sung et al., 2009). Thus, we hypothesized that GATA2 is an intermediate transcript factor regulating KIF20A expression in HBV-related HCC. PROMO database predicted that GATA2 binds to the regulatory sequences in the promoter of KIF20A with 9-bp binding sites (**Figure 6D**). To determine that GATA2 plays a critical role in binding to KIF20A promoter, luciferase reporter constructs containing the wild type or mutant KIF20A promoter sequences were co-transfected with pcDNA3.1/pcDNA-GATA2 into 293T cells (**Figure 6E**). In the GATA2-overexpressed group, the construct containing the wild-type KIF20A promoter sequence showed the highest increase in relative luciferase activity (p = 0.023, **Figure 6F**). However, the activity of the mutant type KIF20A reporter construction was reduced by 3.5-folds, compared to the activity of the wild-type group (p = 0.018, **Figure 6F**). The result showed that GATA2 activated the KIF20A promoter through the critical promoter region between -76 and -68.

### GATA2 drives HBV-related HCC cell proliferation and apoptosis by regulating KIF20A

**Figure 7:**
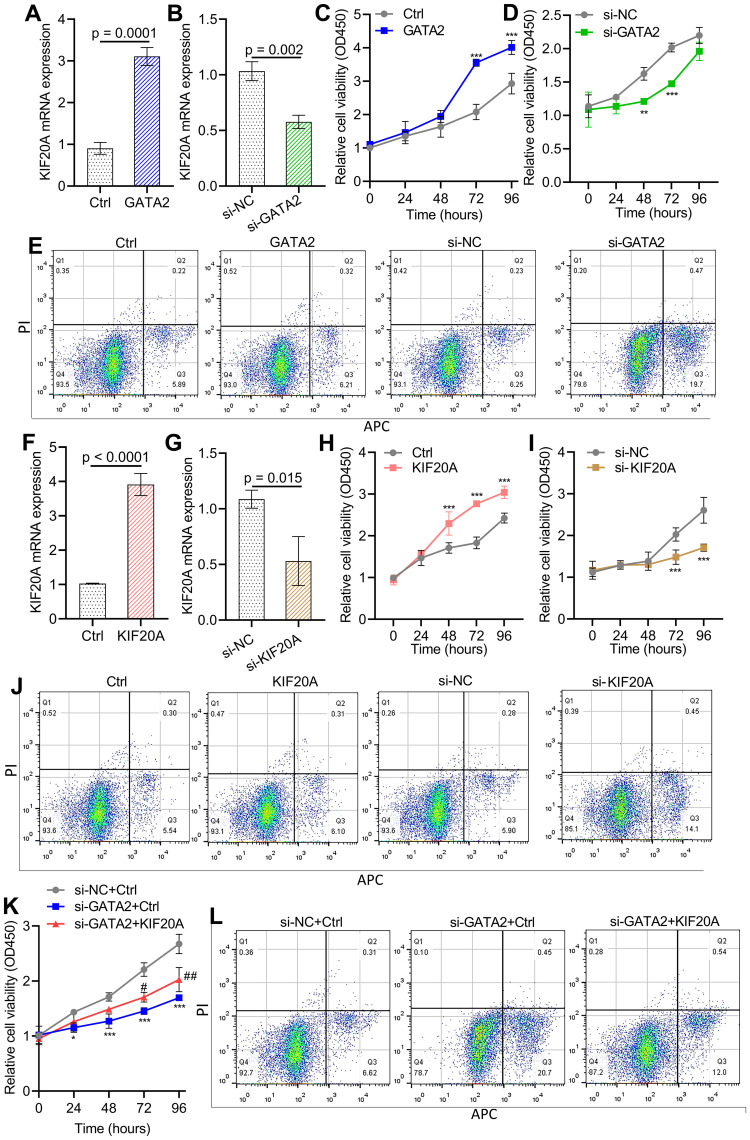
GATA2 promotes HBV-related HCC cell growth and inhibits apoptosis by modulating KIF20A expression **(A, B)** qPCR was utilized to detect the efficiency of GATA2 overexpression and GATA2 silencing. **(C, D)** The effect of GATA2 overexpression and GATA2 silencing on cell growth was detected by CCK-8assay in HepG2.2.15 cells. **(E)** Flow cytometry was utilized to explore the effect of overexpression and silencing GATA2 on HepG2.2.15 cell apoptosis. **(F, G)** The efficiency of KIF20A overexpression and silencing was evaluated using qPCR. **(H-I)** The impact of overexpression and silencing KIF20A on cell growth was assessed using the CCK-8 assay in HepG2.2.15 cells. **(J)** Flow cytometry was utilized to detect the effect of overexpression and silencing KIF20A on HepG2.2.15 cell apoptosis. **(K)** HepG2.2.15 cells were transfected with si-NC/si-GATA2 and ctrl/KIF20A in combination for 48 hours. Cell proliferation was analyzed using a CCK-8 assay. **(L)** Cell apoptosis was determined by flow cytometry. * p < 0.05, ** p < 0.01, ***p < 0.001; # p < 0.05, ## p < 0.01.

To evaluate the potential role of GATA2 in the pathogenesis of HBV-related HCC, we created siRNA molecules and overexpression plasmids targeting GATA2, and our data demonstrated that these constructs significantly impacted KIF20A expression levels in HepG2.2.15 cells (p < 0.001, **Figures 7A, B**). **Supplementary Figure S2** showed the silencing efficiency of siRNAs (all p < 0.001). By performing the CCK-8 cell proliferation assay, we found that HepG2.2.15 cell proliferation was remarkably increased by GATA2 overexpression at 72-96 hours (p < 0.001, **Figure 7C**), but reduced after GATA2 silencing (p < 0.001, **Figure 7D**). Flow cytometry showed that silencing GATA2 promoted HepG2.2.15 cell apoptosis (20.17% vs 6.11%, **Figure 7E**). Inversely, the apoptotic rate of GATA2 overexpression group in HepG2.2.15 cells was similar with the untreated group (6.53% vs 6.48%, **Figure 7E**). Additionally, **Figures 7F–J** also demonstrated the importance of KIF20A in promoting HCC cell growth and inhibiting cell apoptosis. Furthermore, KIF20A reversed the effect of GATA2 interference on HepG2.2.15 cell growth (p < 0.05, p < 0.01, p < 0.001, **Figures 7K, L**).

## Discussion

The current study underscored the importance of the GATA2/KIF20A axis in modulating HCC growth and progression in response to HBV infection, and demonstrated the therapeutic potential of targeting this axis to inhibit HCC cell proliferation.

We undertook comprehensive RNA-sequencing analysis and interrogated the data in relation to KIF20A expression and clinical characteristics of patients with HBV-related HCC, such as overall survival and pathologic stages. Importantly, we firstly observed that KIF20A is highly expressed in HBV-related HCC patients at diagnosis and is associated with worse overall survival. Our results seem to be in accordance with previous investigations reporting an association between KIF20A expression and advanced pathological parameters in bladder cancer and gastric cancer (Sheng et al., 2018; Shen et al., 2019). Furthermore, the gene correlation expression analysis unveiled several genes correlated with the KIF20A expression. For instance, BUB1B, a crucial serine/threonine kinase in the mitotic checkpoint pathway, acts as an oncogenic role in HCC tumor growth and metastasis via activating mTORC1 and JAK-STAT signaling pathways (Qiu et al., 2020; Hamdy et al., 2023). KIF11 is a novel kinesin superfamily that influences childhood lymphoblastic leukemia and mediates cell cycle and migratory capacity in tumor cells (Zhu et al., 2023). It has been reported that KIF11 is involved in the G2/M phase transition and promotes tumor progression by regulating cell cycle checkpoints (Zhu et al., 2023). Another KIF20A co-expressed gene, CCNA2, its overexpression in glioma results in a significant cell proliferation rate and activate G2M checkpoint pathways (Zhou et al., 2024). As for DLGAP5, there is growing evidence to show that overexpression of DLGAP5 promotes breast cell proliferation, migration and cell cycle by activating the JAK2/STAT3 signaling pathway (Li et al., 2023). Moreover, DLGAP5 knockdown significantly inhibited cell proliferation and PLK1 expression in lung adenocarcinoma (Chen et al., 2024). Herein, we have shown that KIF20A is co-expressed with several genes, such as BUB1B, CDK1, KIF11, CCNA2 and DLGAP5. KIF20A co-expressed genes can be detected in regulating PLK1 pathway and HCC cell cycle, highlighting the positive impact of KIF20A in HCC progression (Tian et al., 2020). It will be of great interest to investigate the potential role of KIF20A and its co-expressed genes in HCC cellular behaviors

HBx is a multifunctional protein that interacts with various cellular proteins and pathways, leading to the disruption of normal cellular functions and the promotion of tumorigenesis (Slagle et al., 2015; Sivasudhan et al., 2022). For example, it has been demonstrated that the HBx protein was involved in the regulation of protein expression levels through the ubiquitin proteasome system (UPS), specifically affecting proteins involved in HCC cell cycle and DNA repair (Minor and Slagle, 2014). Specifically, HBx protein is thought to interact with transcription factors and regulate gene expression in HCC, thereby contributing to HCC progression (Sung et al., 2009) (Lee and Lee, 2007). For instance, HBx has been found to activate and modulate transcription factors, like NF-κB (Lim et al., 2013), AP-1 (Benn et al., 1996), and p53 (Choi et al., 2002), which are crucial in controlling inflammation, cell proliferation and apoptosis of HCC cells. Here, we show that HBx overexpression induces an upregulation of oncogene KIF20A, which is followed by HCC cell growth and apoptosis inhibition. This demonstrates that HBx might enhance KIF20A expression via modulating a transcriptional factor in HCC.

Using computational-based searches, PROMO predicted the potential binding sites of GATA2 on KIF20A promoter DNA sequences. Coupled with previous finding, Sung et al. indicated a significant SIWTF score of HBx on GATA2 transcription factor binding sites (Sung et al., 2009). We suspect that HBx might directly interact with GATA2 and then up-regulate KIF20A to promote HCC cell proliferation. In this study, through luciferase reporter assay, we found that GATA2 binds to the KIF20A promoter region. GATA2, a zinc finger transcription factor, plays a role by influencing melanoma biology and lymphatic angiogenesis (Hsu et al., 2015; Nguyen et al., 2018). Chen et al. also established that HBx protein activates ST2 promoter via interacting with GATA2 and then upregulates Th2 cell function and cellular immune function in HCC cells (Chen et al., 2020). Contrary to other study reporting that GATA2 is downregulated in HCC tissues and cell line (Li et al., 2014), our data demonstrated that GATA2 overexpression in HBV-infected HCC cells resulted in a significant increase in cell proliferation. Importantly, strong exogenous KIF20A expression rescued the growth inhibition triggered by silencing GATA2 in HepG2.2.15 cells.

This present research has several limitations. Its retrospective nature, utilizing clinical samples collected from TCGA-LIHC and GEO datasets, might ignore some key clinical information. Due to the longtime of follow-up investigation, we did not collect sufficient clinical samples to validate the prognostic value of KIF20A in HBV-related HCC. In addition, the effects of KIF20A overexpression on PLK1 pathway and cell cycle in HBV-related HCC need to be further studied.

In summary, our study provides a comprehensive understanding of the role of KIF20A in HBV-related HCC. We further explored the upstream regulatory mechanisms of KIF20A and identified HBx protein and GATA2 as key drivers of KIF20A expression, which promotes HCC cell growth. This study provides novel mechanistic insights into the functional interplay between GATA2 and KIF20A, and highlights the potential therapeutic and prognostic value of KIF20A in HBV-related HCC.

## Data Availability

The original contributions presented in the study are included in the article/**Supplementary Material**. Further inquiries can be directed to the corresponding authors.
