# Supplementary figures and images for: KIF20A activated by transcription factor GATA2 promotes cell growth in hepatitis B virus-related hepatocellular carcinoma
Source: Front Cell Infect Microbiol. 2024 Nov 18;14:1497427. doi: 10.3389/fcimb.2024.1497427 (PMC11609149; doi:10.3389/fcimb.2024.1497427)

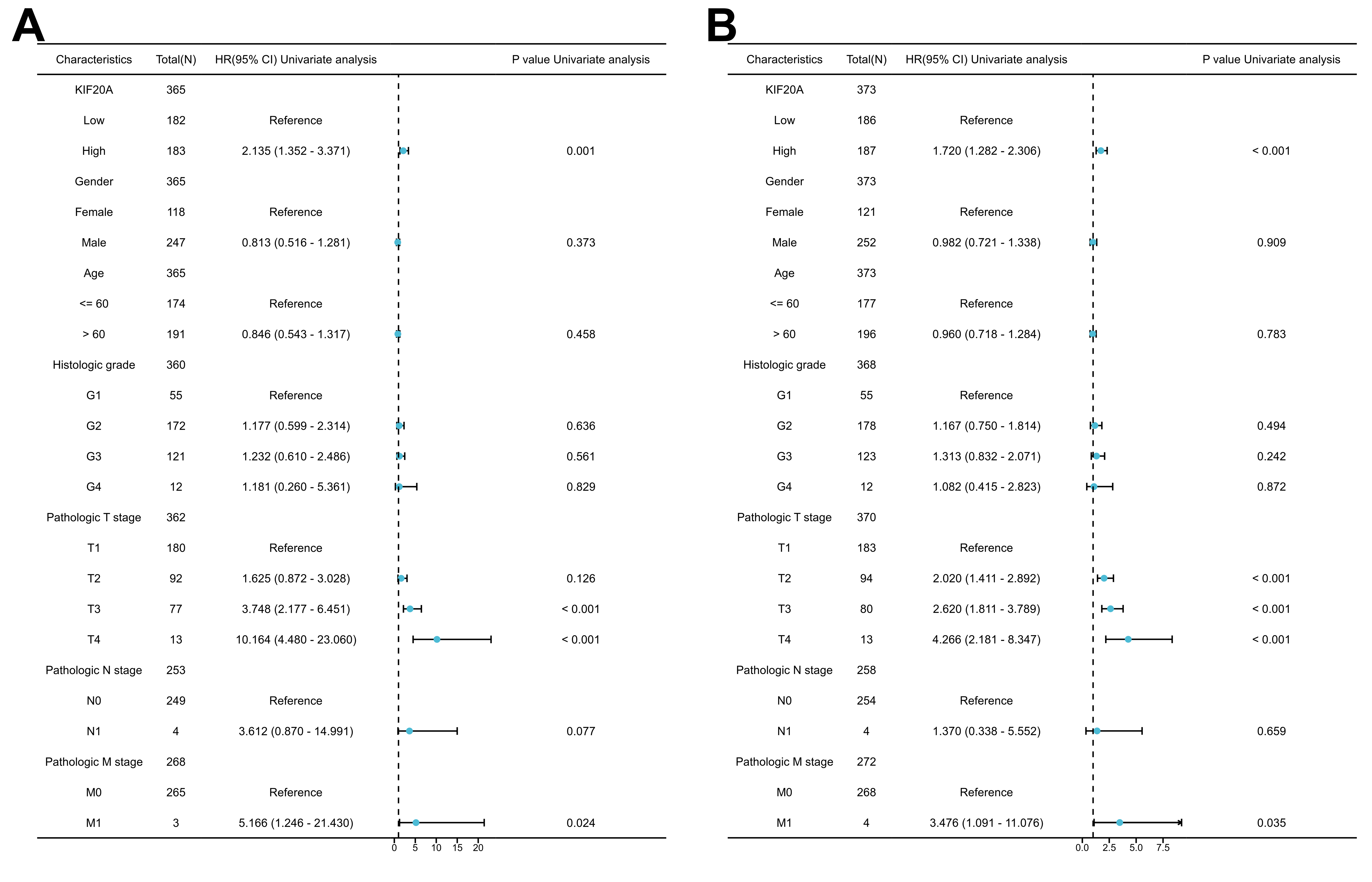

Supplement: Supplementary file 1 [file Image1.tif]

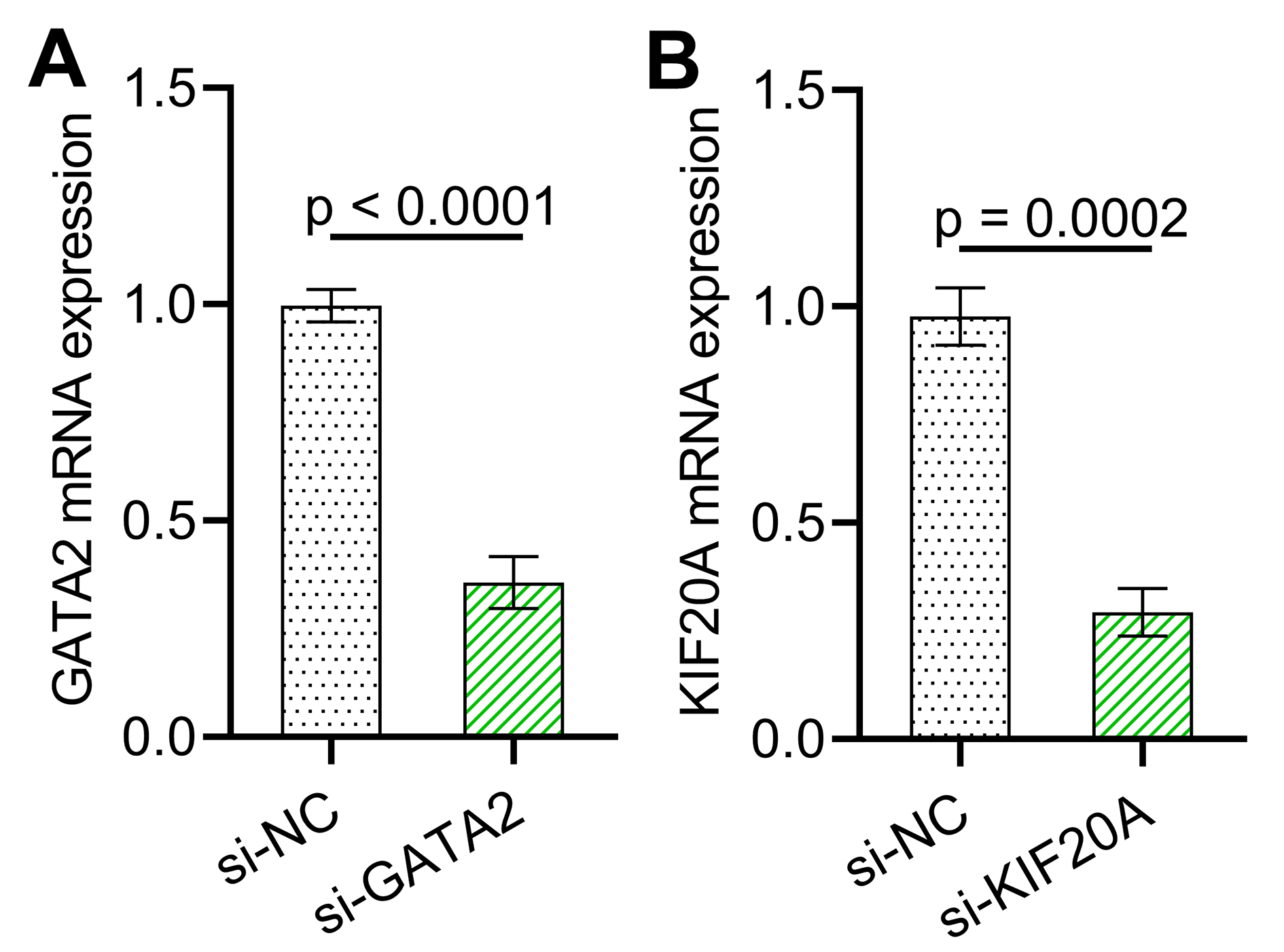

Supplement: Supplementary file 2 [file Image2.tif]
